# Opioid-Free Anesthesia in Perioperative Care: Findings From a Swedish Web-Based Survey

**DOI:** 10.1155/prm/6677904

**Published:** 2025-11-20

**Authors:** Salwan Diwan, Alexander Olausson, Paulin Andréll, Axel Wolf, Pether Jildenstål

**Affiliations:** ^1^Institute of Health and Care Sciences, University of Gothenburg, Sahlgrenska Academy, Gothenburg, Sweden; ^2^Department of Anaesthesiology and Intensive Care/Pain Centre, Region Västra Götaland, Sahlgrenska University Hospital, Gothenburg, Sweden; ^3^Department of Anaesthesiology and Intensive Care, Institute of Clinical Sciences, University of Gothenburg, Sahlgrenska Academy, Gothenburg, Sweden; ^4^University of Gothenburg Centre for Person-Centred Care (GPCC), University of Gothenburg, Sahlgrenska Academy, Gothenburg, Sweden; ^5^Department of Anaesthesiology and Intensive Care, Region Västra Götaland, Sahlgrenska University Hospital, Gothenburg, Sweden; ^6^Faculty of Health Sciences, Department of Nursing and Health Promotion, Oslo Metropolitan University, Oslo, Norway; ^7^Department of Health Sciences, Lund University, Lund, Sweden; ^8^Department of Anaesthesiology, Surgery and Intensive Care Medicine, Region Västra Götaland, Sahlgrenska University Hospital, Gothenburg, Sweden; ^9^Faculty of Nursing and Health Sciences, Nord University, Bodø, Norway; ^10^Department of Anaesthesiology and Intensive Care, Skane University Hospital, Lund, Sweden; ^11^Department of Anaesthesiology and Intensive Care, School of Medical Sciences, Örebro University Hospital, Örebro, Sweden

**Keywords:** attitudes, healthcare professionals, knowledge, opioid-free anesthesia, practices, web-based survey

## Abstract

**Objectives:**

Opioid-free anesthesia (OFA) is a promising alternative to traditional opioid-based anesthesia. Research indicates that OFA reduces postoperative opioid consumption and related adverse effects while maintaining effective pain control and patient safety. Despite these benefits, clinical adoption of OFA remains limited, possible due to gaps in evidence. To bridge this knowledge gap, a web-based survey was used to assess Swedish healthcare professionals' (HCPs') knowledge, attitudes, and practices regarding OFA, aiming to identify barriers and facilitators for its broader integration into routine anesthesia care.

**Methods:**

A web-based survey was sent to anesthesiology department heads at all university hospitals in Sweden and three randomly selected smaller hospitals. The department heads distributed the survey to anesthesiologists, nurse anesthetists, critical care nurses, and registered nurses involved in intraoperative care. The questionnaire had four sections: demographic data, general questions regarding intraoperative care, specific questions on OFA usage, and an open-ended question.

**Results:**

A total of 309 HCPs responded to the survey, corresponding to a response rate of 35%. The majority of respondents (77%) had seven or more years of perioperative experience, 63% were female, and 82% worked at university hospitals. Knowledge about OFA was generally low, with 62% reporting insufficient knowledge, and 14% actively applied OFA. Self-report data demonstrated that anesthesiologists had significantly lower knowledge levels on applying OFA compared to nurse anesthetists and critical care nurses (*p* < 0.01). However, 85% of all HCPs expressed interest in acquiring additional theoretical knowledge about OFA. Additionally, 87% reported either absent guidelines or uncertainty about their existence, while most (57%) agreed that guidelines supporting the practice of OFA should be introduced at their clinic.

**Conclusion:**

This survey indicated interest in OFA among Swedish perioperative HCPs while revealing key barriers to implementation, including knowledge gaps and limited guidelines. Addressing these challenges through targeted education and institutional support may facilitate broader OFA adoption, enhancing patient safety and multimodal perioperative pain management.

## 1. Introduction

In recent years, the demand for and awareness of opioid-free alternatives has grown as research across various disciplines, including oncology, has indicated that opioids may negatively impact survival in certain cancer types [1, 2]. Despite this, opioids remain an essential component of modern anesthesia due to their potent analgesic and sympatholytic effects [3]. However, concerns about opioid-induced adverse events, including postoperative nausea and vomiting (PONV), respiratory compromise, constipation, and sedation, have prompted a reappraisal of their routine use [4–7]. Opioid-induced hyperalgesia, which may require increased opioid dosages for effective postoperative pain management [8], has further contributed to this reassessment. Furthermore, opioids are associated with heightened healthcare costs and an increased likelihood of chronic pain and dependence [9–11].

In response, opioid-free anesthesia (OFA) has gained prominence as a strategy that replaces opioids with medications such as *α*_2*A*_-adrenergic agonists, N-methyl-D-aspartate (NMDA)-receptor antagonists, local anesthetics, magnesium, and beta-blockers [4, 12–16]. A meta-analysis showed that OFA can significantly reduce postoperative adverse events and overall opioid consumption without compromising patient-reported pain control or safety [8]. Certain patient groups, including females, individuals with chronic pain, and those with a history of substance misuse, appear to particularly derive benefit from OFA due to a reduced risk of opioid-related complications [17–19]. A recent randomized controlled trial found that combined intraoperative OFA with postoperative first-line transcutaneous electrical nerve stimulation (TENS) achieved similar pain relief and recovery outcomes to opioid-based standard care. In addition, the combination also significantly reduced in-hospital opioid use, which supports the feasibility of a fully opioid-free pathway in bariatric surgery [20].

Despite growing evidence supporting OFA, its routine clinical adoption remains limited, partly because many clinicians perceive opioids as superior or regard the evidence for nonopioid strategies as insufficient [21, 22]. Additional barriers are embedded within organizational and cultural norms, as well as among patients who frequently view opioids as the “gold standard” for immediate pain relief [23, 24].

Although the potential benefits of OFA are increasingly recognized, no study has systematically examined healthcare professionals' (HCPs') knowledge, attitudes, and practices (KAPs) regarding OFA. Therefore, this web-based survey aims to assess these factors within a Swedish context to promote the integration of OFA into routine clinical practice.

## 2. Methods

A web-based survey was chosen for its efficiency in collecting data from a large and geographically dispersed sample while ensuring participant anonymity and convenience. In October 2023, an email containing an information letter describing the project and a link to the survey was sent to the heads of anesthesiology departments at all university hospitals in Sweden (*n* = 7). Additionally, three smaller hospitals were randomly selected, from a list of hospitals that expressed interest after receiving the information letter, from each of the country's three major geographical areas: Northern Sweden (north of Uppsala), Central Sweden (between Uppsala and Linköping), and Southern Sweden (south of Linköping). Their names were drawn by Alexander Olausson from a bowl to ensure an unbiased selection. Department heads served as gatekeepers for survey distribution. By leveraging their leadership roles, they facilitated internal communication and ensured that the survey reached anesthesiologists, nurse anesthetists, critical care nurses, and registered nurses with basic education. The survey remained open until January 2024, with email reminders sent after 4 and 8 weeks to increase response rates.

The email addresses of the department heads were obtained from Sweden's national healthcare guide website (https://www.1177.se) [25]. The survey was administered via the Qualtrics platform (version 2023-10-2024-01), licensed by the University of Gothenburg. Participation was voluntary, and all respondents provided informed consent before completing the survey anonymously.

### 2.1. Development and Structure of the Questionnaire

A structured, self-report questionnaire was designed to evaluate the KAP of HCPs regarding OFA in perioperative settings. As no previously validated instrument was precisely aligned with the objectives of this study, a self-designed questionnaire was developed based on the KAP model [26]. The same questionnaire, administered during a single data-collection period, also included a separate postoperative section with focus on TENS for acute postoperative pain management, the findings of which have already been published [27].

This questionnaire underwent pilot testing by seven nurses and five anesthesiologists to refine its language and format based on their feedback before data collection commenced. A linguistically verified English questionnaire version is available as a Supporting File 1. OFA questionnaire.

The questionnaire comprised the following sections:

1. Demographic information: Collected data on respondents' age, sex, profession, and years of clinical experience.
2. General questions on intraoperative care: These questions aimed to capture respondents general practices and protocols in the intraoperative setting.
3. Specific questions on OFA usage.
4. Open-ended question: “Is there anything more you would like to add?”

Respondents answered questions using a four-point Likert scale (strongly disagree, disagree, agree, strongly agree) along with an additional “Don't know” option as a separate nominal category. For select questions, respondents were given two or three options to choose from (yes, no, or Don't know).

### 2.2. Analysis

All statistical analyses were conducted using IBM SPSS Statistics [Version 29.0.2.0 [20]]. Demographic data are presented as frequencies and percentages, while survey responses are expressed as percentages, calculated by dividing the number of positive or negative responses by the total number of responses for each question. Kruskal–Wallis tests assessed significant differences across demographic subgroups for each questionnaire item. A *p*-value < 0.05 was considered indicative of statistical significance. When significant differences were detected, post hoc analyses using the Bonferroni correction were applied to adjust for multiple comparisons. Because the variable “sex” included two groups (male and female), Mann–Whitney *U*-tests were employed as post hoc tests using the results from the Kruskal–Wallis tests of significance. Detailed results of these analyses are presented in Appendix Tables A1, A2, A3.

## 3. Results

Based on feedback from the department heads, the survey was distributed to 870 HCPs, yielding 309 responses (35% response rate). Among the respondents, over 77% had at least 7 years of experience in perioperative practice, 63% were female, 70% were 41 years or older, and 82% worked at a university hospital. The demographics of the respondents are presented in Table 1.

### 3.1. Knowledge of OFA

**Figure 1:**
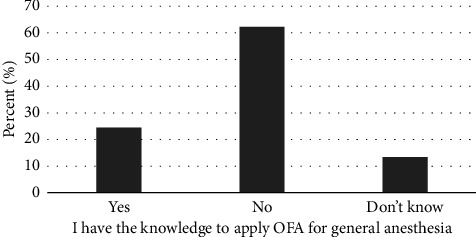
Respondent's knowledge of applying opioid-free anesthesia (OFA) in general anesthesia.

Among the respondents, 62% reported lacking the required knowledge to apply OFA in general anesthesia (Figure 1): 24% reported having the required knowledge, which was acquired through various sources: university lectures (5%), scientific articles (13%), workplace lectures (10%), clinical practice (8% weekly, 3% monthly, 5% annually), and other sources (3%).

Knowledge of OFA application varied significantly by sex (H1 = 12.3, *p* < 0.01) and profession (H3 = 29.1, *p* < 0.01). Females reported higher knowledge of OFA for general anesthesia than males (mean rank of 143.6 and 117.0, respectively; see Table A3, Q. 3.1). Anesthesiologists reported significantly lower knowledge levels than nurse anesthetists (adjusted *p* = 0.01) and critical care nurses (adjusted *p* < 0.01). Although anesthesiologists reported lower knowledge levels than registered nurses with basic education (*p* = 0.01), this difference was not statistically significant (adjusted *p* = 0.01). No significant differences were observed across age, workplace, or years of experience (see Tables A1 and A2, Q. 3.1).

### 3.2. Attitudes Toward OFA

**Figure 2:**
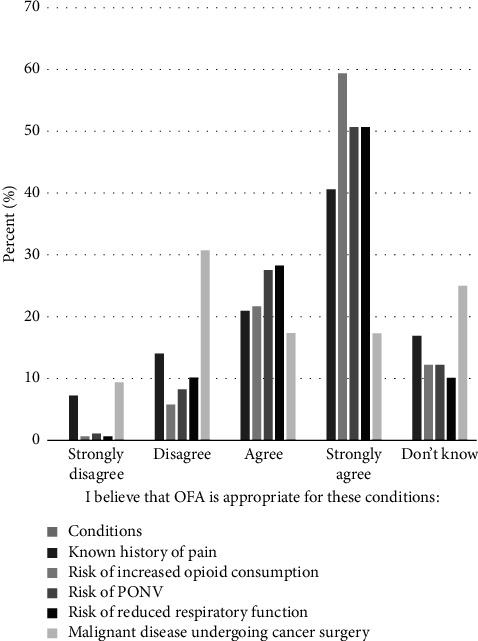
Perceptions of opioid-free anesthesia appropriateness for patients undergoing surgery.

When asked about the appropriateness of OFA for various conditions, the respondents provided the following:

For patients at risk for or with known history of pain, 69% considered OFA appropriate or very appropriate (Figure 2). A significant variation was observed based on age (H4 = 11.4, *p* = 0.02); however, no statistically significant differences were detected between specific age groups. No significant differences were observed across sex, profession, workplace, or years of experience (see Tables A1 and A2, Q 3.2.1).

A proportion of 75% of respondents agreed or strongly agreed that OFA was suitable for patients at risk for increased opioid consumption or misuse. A significant variation across professions was observed (H3 = 8.0, *p* = 0.05). Specifically, the suitability of OFA was rated significantly higher by critical care nurses than by nurse anesthetists (*p* = 0.01). However, this difference was not significant after adjustment (*p* = 0.07). No significant differences were observed across sex, age, workplace, or years of experience in perioperative practice (see Tables A1 and A2, Q. 3.2.2).

For patients at risk for PONV, 73% of respondents agreed or strongly agreed that OFA is suitable for this patient group. Significant variations based on sex and profession were observed (H1 = 4.9, *p* = 0.03 and H3 = 8.2, *p* = 0.04, respectively). Females reported stronger agreement with the suitability of OFA for PONV compared to males (mean rank 128.5 compared to 110.6, respectively; see Table A3, Q. 3.2.3). Critical care nurses and nurse anesthetists also showed significant differences (*p* = 0.01), although this difference was no longer significant after adjustment (*p* = 0.06). Likewise, the significant difference between anesthesiologists and nurse anesthetists (*p* = 0.04) was also no longer significant after adjustment (*p* = 0.24). No significant differences were observed based on age, occupational place, or years of experience in perioperative practice (see Tables A1 and A2, Q. 3.2.3).

A total of 68% of respondents agreed or strongly agreed that OFA was suitable for patients at risk for reduced postoperative respiratory function. Significant differences were observed for sex (H1 = 6.9, *p* = 0.01) and experience in perioperative practice (H4 = 10.1, *p* = 0.04). Females reported stronger agreement with the suitability of OFA for this condition compared to males (mean rank of 133.0 and 111.3, respectively; see Table A3, Q. 3.2.4). Respondents with 12–14 years of experience showed an initial significant difference compared to those with 7–11 years of experience (*p* = 0.01); however, this difference did not remain significant after adjustment (*p* = 0.12). Similarly, those with 12–14 years of experience significantly differed from those with 3–6 years of experience (*p* = 0.01); however, this difference was also not significant after adjustment (*p* = 0.11). No significant differences were observed based on age, profession, or occupational place (see Tables A1 and A2, Q. 3.2.4).

A total of 35% of HCPs agreed or strongly agreed that OFA was suitable for patients with malignant disease undergoing cancer surgery, while 31% disagreed. No significant variations were found for sex, age, profession, workplace, or experience in perioperative practice (see Tables A1 and A2, Q. 3.2.5).

**Figure 3:**
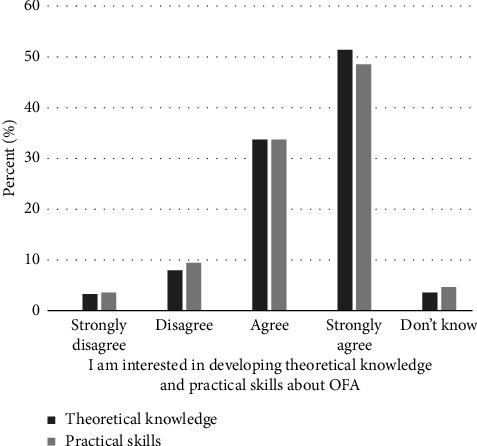
Interest in developing theoretical knowledge and practical skills in opioid-free anesthesia (OFA).

A proportion of 85% of respondents agreed or strongly agreed that they were interested in developing theoretical knowledge about OFA (Figure 3). Sex showed a significant difference for this factor (H1 = 5.5, *p* = 0.02); females reported stronger agreement than males (mean rank of 141.4 and 121.1, respectively; see Table A3, Q.3.3). However, no significant variations were observed based on age, profession, workplace, or experience in perioperative practice (see Tables A1 and A2, Q.3).

A total of 82% of respondents agreed or strongly agreed that they were interested in developing practical skills for the application of OFA. No significant differences were detected based on sex, age, profession, workplace, or experience in perioperative practice (see Tables A1 and A2, Q. 3.4).

### 3.3. Practice of OFA

When asked about their application of OFA for general anesthesia, 86% reported that they did not apply OFA, while 14% reported that they did. No significant variations were observed based on sex, age, profession, workplace, or experience in perioperative practice (see Tables A1 and A2, Q. 3.5).

#### 3.3.1. Guidelines for OFA

When asked whether guidelines were available at their clinic supporting the practice of OFA for general anesthesia, 49% reported that no such guidelines existed, while 38% were unsure, and 13% confirmed the presence of guidelines. No significant differences based on sex, age, profession, workplace, or experience in perioperative practice (see Tables A1 and A2, Q. 3.6).

Among this 13% of respondents who confirmed the presence of guidelines supporting OFA for general anesthesia, 65% expressed satisfaction, either agreeing or strongly agreeing. No significant variation based on sex, age, profession, workplace, or experience in perioperative practice (see Tables A1 and A2, Q. 3.6.1).

Among those who reported a lack of guidelines or were unsure, 57% agreed that guidelines should be introduced at their clinic, 37% were uncertain, and 6.3% disagreed. The analysis showed significant differences based on sex (H1 = 17.4, *p* < 0.01) and profession (H3 = 8.9, *p* = 0.03). Males reported higher agreement about the introduction of guidelines compared to females, as indicated by a mean rank of 84.4 compared to 68.9, respectively (see Table A3, Q. 3.6.2). Although comparisons between professions revealed an initial significant difference between anesthesiologists and nurse anesthetists (*p* = 0.01), this difference did not remain significant after adjustment (*p* = 0.08). No significant variations based on age, occupational place, or years of experience in perioperative practice were observed (see Tables A1 and A2, Q. 3.6.2).

## 4. Discussion

This web-based survey provides a comprehensive overview of Swedish perioperative HCPs' KAP regarding OFA. The findings highlight a notable contrast between generally positive attitudes toward OFA and its limited practical implementation. In contrast, many respondents acknowledged the potential benefits of OFA, such as reduced opioid consumption and fewer opioid-related adverse events; a minority reported routinely incorporating OFA into their clinical practice. A U.S. survey of 2883 Certified Registered Nurse Anesthetists (CRNAs) reported that 81% had administered OFA and 88% viewed it as beneficial [21]. Despite this high reported exposure, barriers such as institutional culture and limited access to multimodal anesthetics were identified. Compared to the 14% usage rate in our sample, these differences may reflect varying national guidelines, training, and organizational support.

Several factors likely contribute to this discrepancy between theoretical support and clinical application. First, limited knowledge and practical experience with OFA were evident among respondents; a few reported familiarity with OFA techniques and fewer had applied OFA in clinical practice. This lack of understanding might have contributed to uncertainty regarding the analgesic efficacy of nonopioid compared to opioid agents. Respondents might had limited exposure to validated OFA protocols or insufficient hands-on experience with specific techniques. Second, the absence or underutilization of institutional OFA guidelines emerged as a frequently cited barrier, mirroring findings from other healthcare systems that emphasize the importance of clear, evidence-based protocols in promoting effective nonopioid pain management. Consistent with previous research [21, 22], our findings suggest that the development and dissemination of institutional protocols, together with targeted education, are essential for enhancing the implementation and acceptance of OFA in clinical practice.

The majority of HCPs agreed or strongly agreed that OFA was suitable for various patient groups with conditions in which the use of opioids might compromise postoperative recovery, such as in patients at risk of PONV or patients at risk of reduced respiratory function. The growing awareness of OFA's potential to enhance recovery after surgery aligns with patients' desire to be actively involved in decisions about postoperative pain management, including access to information on available options, benefits, and risks [28]. This highlights the clinical value of OFA as a means to reduce opioid-related harm, individualize treatment, and support person-centered care by accounting for patient preferences. Nevertheless, many patients continue to perceive opioids as the gold standard for immediate postoperative pain relief, which may present a barrier to clinical implementation [23, 24].

Another key insight from this survey is the influence of professional roles, years of experience, and workplace culture on OFA adoption. While most anesthesiologists and nurse anesthetists demonstrated familiarity with OFA, their actual implementation of OFA protocols depended on local resource availability, including access to specific medications and infusion pumps and institutional readiness to support change. Notably, critical care nurses reported higher knowledge levels despite their limited practical experience with OFA in the context of general anesthesia. However, these responses could reflect theoretical familiarity rather than applied expertise. These findings align with international data showing that organizational constraints, such as drug formularies, staff education, and operating room scheduling, can significantly delay integrating new practices [21–24].

Significant differences were observed between males and females in their knowledge and attitudes toward OFA. Females demonstrated higher knowledge levels and greater interest in developing a theoretical understanding of OFA. Males, on the other hand, reported stronger agreement with the need to implement OFA guidelines at their clinics. These results indicate a need for tailored educational efforts to support the broader implementation of OFA across different professional perspectives.

The strong interest among HCPs in further OFA education and protocol development is an encouraging sign that should be actively utilized and integrated into clinical practice. Their willingness to learn about multimodal analgesic strategies suggests that knowledge gaps could be addressed through targeted educational programs, such as in-house seminars, online modules, or formal workshops. Such initiatives may benefit HCPs with minimal prior experience in OFA. Furthermore, professional associations could play a leading role in developing comprehensive guidelines to address concerns regarding inconsistent or unclear protocols.

### 4.1. Limitations

While the questionnaire was not formally validated using psychometric methods, it was thoroughly pilot tested with both nurses and anesthesiologists to ensure clarity, relevance, and practical applicability. This pragmatic approach allowed the instrument to be tailored to the target population and clinical context. Nevertheless, the absence of formal validation may limit the extent to which the findings can be generalized beyond this setting. Although the response rate of 35% compares favorably with many web-based healthcare surveys, often below 20%, it nonetheless limits the generalizability of our findings and raises the possibility of response bias [29–31]. Despite employing recommended strategies such as direct email invitations, reminders, and a brief survey format [32], some perioperative HCPs may have opted not to participate due to limited interest or time constraints. Conversely, those particularly interested in OFA may have been more inclined to respond, potentially overrepresenting favorable attitudes toward OFA.

Relying on department heads as gatekeepers for survey distribution may have also introduced selection bias, particularly if the survey was emphasized more strongly in certain hospitals, especially university centers with a stronger academic focus on multimodal or innovative analgesic techniques. Consequently, our results may disproportionately reflect the perspectives of clinicians who practice in well-resourced facilities, limiting the transferability of our findings to smaller or resource-limited settings. In addition, as respondents represented different professional groups, their varying roles and responsibilities in anesthesia care may have influenced their perspectives on OFA. These factors should be considered when interpreting the findings in relation to national trends in anesthesia practice.

Additionally, reliance on self-reported data introduces limitations such as social desirability and recall biases. Respondents may have been motivated to present a more proactive stance on OFA due to increasing awareness of opioid-related challenges in perioperative care. Moreover, since the survey included a subset of Swedish hospitals, albeit encompassing all university hospitals and a sample of smaller hospitals across three geographical regions, our results may not capture the full breadth of perioperative practices nationwide.

Future studies should aim for a larger, more diverse sample to strengthen generalizability and reduce the risk of selection bias. Qualitative methods, such as focus groups or interviews, could supplement survey data by providing deeper insights into the institutional, cultural, and individual factors influencing the adoption and sustained use of OFA. Longitudinal or interventional study designs could further clarify whether targeted educational initiatives and updated clinical guidelines improve OFA implementation and patient outcomes.

## 5. Conclusion

This web-based survey provides critical insights into the current landscape of OFA in Sweden, underscoring an interest among perioperative HCPs and the practical barriers hindering widespread implementation. While many HCPs recognize the potential benefits of OFA, namely reduced opioid consumption and fewer opioid-related adverse events, knowledge gaps, limited clinical guidelines, and perceived resource constraints continue to impede its routine adoption. The strong interest among respondents in further education and standardized protocols suggests that targeted educational programs, coupled with robust institutional support, could bridge the gap between theoretical endorsement and practical usage. This way, OFA may evolve from an underutilized alternative to a mainstay of multimodal perioperative pain management, ultimately improving patient safety and long-term outcomes in anesthesia practice.

## Figures and Tables

**Table 1:** Demographic characteristics of the respondents.

Demographic of responders	*n* (%)
Sex	
Male	116 (37.5)
Female	193 (62.5)
Total	309 (100)
Age	
20–30	7 (2.3)
31–40	87 (28.2)
41–50	99 (32.0)
51–60	79 (25.6)
≥ 61	37 (12.0)
Profession	
Anesthesiologist	140 (45.3)
Nurse anesthetist	86 (27.8)
Critical care nurses	73 (23.6)
Registered nurse, basic education	10 (3.2)
Workplace	
University hospital	253 (81.9)
County hospital	47 (15.2)
Other hospital/infirmary	9 (2.9)
Professional experience in perioperative practice	
0–2 years	33 (10.7)
3–6 years	37 (12.0)
7–11 years	67 (21.7)
12–14 years	34 (11.0)
≥ 15 years	138 (44.7)

**Table 2:** Results of Kruskal–Wallis tests comparing demographic subgroups for each questionnaire item, reporting medians, test statistics (H), degrees of freedom (df), and *p* values.

Question	Variable		*N*	Median	Kruskal–Wallis *H*	df	*p* value
Q. 3.1 I have the knowledge to apply OFA for general anesthesia	Sex	Male	101	2	12.317	1	0.001
		Female	165				
		Total	266				
	Age	20–30 years	6	2	6.564	4	0.161
		31–40 years	77				
		41–50 years	80				
		51–60 years	70				
		≥ 61 years	33				
		Total	266				
	Profession	Anesthesiologist	126	2	29.130	3	0.001
		Nurse anesthetist	73				
		Nurse in intensive care	60				
		Registered nurse, basic education	7				
		Total	266				
	Occupational place	University hospital	220	2	0.151	2	0.927
		County hospital	39				
		Other hospital/infirmary	7				
		Total	266				
	Experience in perioperative practice	0–2 years	26	2	6.705	4	0.152
		3–6 years	34				
		7–11 years	59				
		12–14 years	25				
		≥ 15 years	122				
		Total	266				

Q.3.2.1 I believe that OFA is appropriate for patients at risk for or with known history of pain	Sex	Male	91	3	0.345	1	0.557
		Female	138				
		Total	229				
	Age	20–30 years	4	3	11.399	4	0.022
		31–40 years	71				
		41–50 years	69				
		51–60 years	57				
		≥ 61 years	28				
		Total	229				
	Profession	Anesthesiologist	119	3	3.383	3	0.336
		Nurse anesthetist	64				
		Nurse in intensive care	38				
		Registered nurse, basic education	8				
		Total	229				
	Occupational place	University hospital	194	3	1.026	2	0.599
		County hospital	29				
		Other hospital/infirmary	6				
		Total	229				
	Experience in perioperative practice	0–2 years	20	3	6.353	4	0.174
		3–6 years	30				
		7–11 years	55				
		12–14 years	27				
		≥ 15 years	97				
		Total	229				

Q 3.2.2 I believe that OFA is appropriate for patients at risk for increased opioid consumption or misuse	Sex	Male	95	4	3.295	1	0.070
		Female	147				
		Total	242				
	Age	20–30 years	4	4	11.382	4	0.23
		31–40 years	71				
		41–50 years	78				
		51–60 years	61				
		≥ 61 years	28				
		Total	241				
	Profession	Anesthesiologist	126	4	8.015	3	0.046
		Nurse anesthetist	67				
		Nurse in intensive care	41				
		Registered nurse, basic education	8				
		Total	242				
	Occupational place	University hospital	202	4	1.157	2	0.556
		County hospital	32				
		Other hospital/infirmary	8				
		Total	242				
	Experience in perioperative practice	0–2 years	23	4	3.637	4	0.457
		3–6 years	31				
		7–11 years	57				
		12–14 years	27				
		≥ 15 years	104				
		Total	242				

Q 3.2.3 I believe that OFA is appropriate for patients at risk for postoperative nausea and vomiting (PONV)	Sex	Male	95	4	4.900	1	0.027
		Female	147				
		Total	242				
	Age	20–30 years	4	4	3.016	4	0.555
		31–40 years	72				
		41–50 years	78				
		51–60 years	60				
		≥ 61 years	28				
		Total	242				
	Profession	Anesthesiologist	125	4	8.226	3	0.042
		Nurse anesthetist	70				
		Nurse in intensive care	39				
		Registered nurse, basic education	8				
		Total	242				
	Occupational place	University hospital	207	4	2.179	2	0.336
		County hospital	33				
		Other hospital/infirmary	8				
		Total	242				
	Experience in perioperative practice	0–2 years	23	4	3.562	4	0.469
		3–6 years	31				
		7–11 years	57				
		12–14 years	28				
		≥ 15 years	103				
		Total	242				

Q 3.2.4 I believe that OFA is appropriate for patients at risk for reduced postoperative respiratory function	Sex	Male	95	4	6.888	1	0.009
		Female	151				
		Total	248				
	Age	20–30 years	4	4	3.461	4	0.484
		31–40 years	72				
		41–50 years	80				
		51–60 years	62				
		≥ 61 years	30				
		Total	248				
	Profession	Anesthesiologist	128	4	10.539	3	0.14
		Nurse anesthetist	69				
		Nurse in intensive care	43				
		Registered nurse, basic education	8				
		Total	248				
	Occupational place	University hospital	207	4	4.780	2	0.92
		County hospital	33				
		Other hospital/infirmary	8				
		Total	248				
	Experience in perioperative practice	0–2 years	25	4	10.108	4	0.039
		3–6 years	30				
		7–11 years	58				
		12–14 years	29				
		≥ 15 years	106				
		Total	48				

Q 3.2.5 I believe that OFA is appropriate for patients with malignant disease undergoing cancer surgery	Sex	Male	85	2	3.416	1	0.065
		Female	122				
		Total	207				
	Age	20–30 years	4	2	6.190	4	0.185
		31–40 years	60				
		41–50 years	70				
		51–60 years	49				
		≥ 61 years	24				
		Total	207				
	Profession	Anesthesiologist	112	2	5.702	3	0.127
		Nurse anesthetist	53				
		Nurse in intensive care	36				
		Registered nurse, basic education	6				
		Total	207				
	Occupational place	University hospital	174	2	4.741	2	0.093
		County hospital	27				
		Other hospital/infirmary	6				
		Total	207				
	Experience in perioperative practice	0–2 years	23	2	5.394	4	0.249
		3–6 years	26				
		7–11 years	47				
		12–14 years	23				
		≥ 15 years	88				
		Total	207				

Q 3.3 I am interested in developing theoretical knowledge about OFA	Sex	Male	104	4	5.488	1	0.019
		Female	162				
		Total	266				
	Age	20–30 years	5	4	4.053	4	0.399
		31–40 years	76				
		41–50 years	86				
		51–60 years	69				
		≥ 61 years	30				
		Total	266				
	Profession	Anesthesiologist	131	4	3.783	3	0.286
		Nurse anesthetist	74				
		Nurse in intensive care	53				
		Registered nurse, basic education	8				
		Total	266				
	Occupational place	University hospital	221	4	1.875	2	0.392
		County hospital	36				
		Other hospital/infirmary	9				
		Total	266				
	Experience in perioperative practice	0–2 years	26	4	7.494	4	0.112
		3–6 years	34				
		7–11 years	60				
		12–14 years	31				
		≥ 15 years	115				
		Total	266				

Q 3.4 I am interested in developing practical skills about OFA	Sex	Male	104	4	3.625	1	0.057
		Female	159				
		Total	263				
	Age	20–30 years	5	4	7.623	4	0.106
		31–40 years	74				
		41–50 years	86				
		51–60 years	69				
		≥ 61 years	29				
		Total	263				
	Profession	Anesthesiologist	131	4	4.836	3	0.184
		Nurse anesthetist	74				
		Nurse in intensive care	51				
		Registered nurse, basic education	7				
		Total	263				
	Occupational place	University hospital	218	4	0.678	2	0.713
		County hospital	36				
		Other hospital/infirmary	9				
		Total	263				
	Experience in perioperative practice	0–2 years	26	4	9.515	4	0.49
		3–6 years	34				
		7–11 years	58				
		12–14 years	31				
		≥ 15 years	114				
		Total	263				

Q 3.5 I apply OFA for general anesthesia	Sex	Male	106	2	3.030	1	0.082
		Female	168				
		Total	274				
	Age	20–30 years	5	2	1.443	4	0.837
		31–40 years	78				
		41–50 years	86				
		51–60 years	73				
		≥ 61 years	32				
		Total	274				
	Profession	Anesthesiologist	132	2	5.339	3	0.149
		Nurse anesthetist	74				
		Nurse in intensive care	58				
		Registered nurse, basic education	10				
		Total	274				
	Occupational place	University hospital	227	2	0.606	2	0.739
		County hospital	38				
		Other hospital/infirmary	9				
		Total	274				
	Experience in perioperative practice	0–2 years	28	2	3.350	4	0.501
		3–6 years	35				
		7–11 years	61				
		12–14 years	31				
		≥ 15 years	119				
		Total	274				

Q 3.6 There are guidelines at my clinic supporting the practice of OFA for general anesthesia	Sex	Male	73	2	0.452	1	0.502
		Female	98				
		Total	171				
	Age	20–30 years	3	2	3.305	4	0.508
		31–40 years	49				
		41–50 years	61				
		51–60 years	42				
		≥ 61 years	16				
		Total	171				
	Profession	Anesthesiologist	100	2	1.099	3	0.777
		Nurse anesthetist	56				
		Nurse in intensive care	12				
		Registered nurse, basic education	3				
		Total	171				
	Occupational place	University hospital	142	2	1.253	2	0.534
		County hospital	23				
		Other hospital/infirmary	6				
		Total	171				
	Experience in perioperative practice	0–2 years	15	2	3.328	4	0.505
		3–6 years	22				
		7–11 years	38				
		12–14 years	16				
		≥ 15 years	80				
		Total	171				

Q 3.6.1 I am satisfied with the clinic's guidelines supporting OFA for general anesthesia	Sex	Male	12	3	2.580	1	0.108
		Female	17				
		Total	29				
	Age	20–30 years	—	2	4.263	3	0.234
		31–40 years	4				
		41–50 years	10				
		51–60 years	11				
		≥ 61 years	4				
		Total	29				
	Profession	Anesthesiologist	17	3	4.475	2	0.102
		Nurse anesthetist	9				
		Nurse in intensive care	3				
		Registered nurse, basic education	—				
		Total	29				
	Occupational place	University hospital	22	3	0.178	2	0.915
		County hospital	6				
		Other hospital/infirmary	1				
		Total	29				
	Experience in perioperative practice	0–2 years	—	3	4.092	3	0.252
		3–6 years	3				
		7–11 years	3				
		12–14 years	4				
		≥ 15 years	19				
		Total	29				

Q 3.6.2 Guidelines regarding OFA for general anesthesia should be implemented in my clinic	Sex	Male	64	1	17.374	1	0.001
		Female	86				
		Total	150				
	Age	20–30 years	4	1	1.940	4	0.747
		31–40 years	57				
		41–50 years	44				
		51–60 years	32				
		≥ 61 years	12				
		Total	150				
	Profession	Anesthesiologist	75	1	8.990	3	0.029
		Nurse anesthetist	44				
		Nurse in intensive care	26				
		Registered nurse, basic education	5				
		Total	150				
	Occupational place	University hospital	125	1	0.469	2	0.791
		County hospital	21				
		Other hospital/infirmary	4				
		Total					
	Experience in perioperative practice	0–2 years	18	1	7.811	4	0.099
		3–6 years	24				
		7–11 years	40				
		12–14 years	16				
		≥ 15 years	52				
		Total	150				

**Table 3:** Results of post hoc pairwise comparisons (Bonferroni-corrected) for variables with significant Kruskal–Wallis tests, reporting between-group contrasts, test statistics, standard errors, and adjusted *p* values.

Question	Variable	Sample 1-Sample 2	Test statistic	Std. error	Std. test statistic	Sig.	Adj. sig. (a)
Q. 3.1 I have the knowledge to apply OFA for general anesthesia	Profession	Anesthesiologist—nurse anesthetist	−27.849	8.819	−3.158	0.002	0.010
		Anesthesiologist—critical care nurses	−45.917	9.404	−4.882	< 0.001	0.000
		Anesthesiologist—registered nurse, basic education	−57.000	23.282	−2.448	0.014	0.086
		Nurse anesthetist—critical care nurses	−18.067	10.448	−1.729	0.084	0.503
		Nurse anesthetist—registered nurse, basic education	−29.151	23.723	−1.229	0.219	1.000
		Critical care nurses—registered nurse, basic education	−11.083	23.947	−0.463	0.643	1.000

Q 3.2.1 I believe that OFA is appropriate for patients at risk for or with known history of pain	Age	51–60 years-3–40 years	—			—	—
		41–50 years-20–30 years	—			—	—
		51–60 years-20–30 years	4.724			0.030	0.298
		≥ 61 years-20–30 years	7.619			0.006	0.058
		41–50 years-31–40 years	—			—	—
		51–60 years-≥ 61 years	1.848			0.174	1.000
		≥ 61 years-31–40 years	6.838			0.009	0.089
		41–50 years-51–60 years	0.368			0.544	1.000
		41–50 years-≥ 61 years	3.478			0.062	0.622
		20–30 years-31–40 years	—			—	—

Q 3.2.2 I believe that OFA is appropriate for patients at risk for increased opioid consumption or misuse	Profession	Critical care nurses—anesthesiologist	14.504	10.327	1.404	0.160	0.961
		Critical care nurses—nurse anesthetist	28.516	11.389	2.504	0.012	0.074
		Critical care nurses—registered nurse, basic education	−41.829	22.201	−1.884	0.060	0.357
		Anesthesiologist—nurse anesthetist	−14.012	8.685	−1.613	0.107	0.640
		Anesthesiologist—registered nurse, basic education	−27.325	20.943	−1.305	0.192	1.000
		Nurse anesthetist—registered nurse, basic education	−13.313	21.486	−0.620	0.535	1.000

Q 3.2.3 I believe that OFA is appropriate for patients at risk for postoperative nausea and vomiting (PONV)	Profession	Critical care nurses—anesthesiologist	12.788	11.300	1.132	0.258	1.000
		Critical care nurses-nurse anesthetist	31.743	12.311	2.579	0.010	0.060
		Critical care nurses—registered nurse, basic education	−34.748	23.912	−1.453	0.146	0.877
		Anesthesiologist—nurse anesthetist	−18.955	9.197	−2.061	0.039	0.236
		Anesthesiologist—registered nurse, basic education	−21.961	22.469	−0.977	0.328	1.000
		Nurse anesthetist—registered nurse, basic education	−3.005	22.993	−0.131	0.896	1.000

Q 3.2.4 I believe that OFA is appropriate for patients at risk for reduced postoperative respiratory function	Experience in perioperative practice	12–14 years-≥ 15 years	−16.708	13.341	−1.252	0.210	1.000
		12–14 years-0–2 years	20.353	17.374	1.171	0.241	1.000
		12–14 years-7–11 years	36.466	14.479	2.519	0.012	0.118
		12–14 years-3–6 år	41.926	16.578	2.529	0.011	0.114
		≥ 15 years-0–2 years	3.645	14.154	0.258	0.797	1.000
		≥ 15 years-7–11 years	19.757	10.398	1.900	0.057	0.574
		≥ 15 years-3–6 years	25.218	13.165	1.915	0.055	0.554
		0–2 years-7–11 years	−16.112	15.231	−1.058	0.290	1.000
		0–2 years-3–6 years	−21.573	17.240	−1.251	0.211	1.000
		7–11 years-3–6 years	5.461	14.317	0.381	0.703	1.000

Q 3.6.2 Guidelines regarding OFA for general anesthesia should be implemented in my clinic	Profession	Anesthesiologist—nurse anesthetist	6.059			0.014	0.083
		Anesthesiologist—critical care nurses	2.941			0.086	0.518
		Anesthesiologist—registered nurse, basic education	1.035			0.309	1.000
		Nurse anesthetist—critical care nurses	0.146			0.703	1.000
		Nurse anesthetist—registered nurse, basic education	0.116			0.733	1.000
		Critical care nurses—registered nurse, basic education	0.199			0.656	1.000

**Table 4:** Post hoc results for the variable “sex” (male vs. female), reporting mean ranks, Mann–Whitney *U*, *Z*, and two-tailed *p* values.

Question	Variable	Mean rank (male)	Mean rank (female)	Mann–Whitney *U*	*Z*	*p* value (2-tailed)
Q. 3.1 I have the knowledge to apply OFA for general anesthesia	Sex	117.01	143.59	6667.000	−3.510	< 0.001
Q 3.2.3 I believe that OFA is appropriate for patients at risk for postoperative nausea and vomiting (PONV)	Sex	110.59	128.55	5946.500	−2.214	0.027
Q 3.2.4 I believe that OFA is appropriate for patients at risk for reduced postoperative respiratory function	Sex	111.26	133.00	6039.500	−2.624	0.009
Q 3.3 I am interested in developing theoretical knowledge about OFA	Sex	121.13	141.44	7137.500	−2.343	0.019
Q 3.6.2 Guidelines regarding OFA for general anesthesia should be implemented in my clinic	Sex	84.41	68.87	2182.000	−4.168	< 0.001

## Data Availability

The data that support the findings of this study are available from the corresponding author upon reasonable request.

## Appendix A1

This appendix presents the outcomes of Kruskal–Wallis tests performed to assess significant differences across demographic subgroups for each questionnaire item, including medians, test statistics, degrees of freedom, and *p*-values (see Table A1).

## Appendix A2

This appendix presents results of post hoc analyses using the Bonferroni correction for variables with significant Kruskal–Wallis results, including comparisons between groups, test statistics, standard errors, and adjusted significance levels (see Table A2).

## Appendix A3

This appendix presents post hoc test results specifically for the variable “sex,” comparing male and female responses, including mean ranks, *U*-values, Z-scores, and two-tailed *p*-values (see Table A3).
