# Supplementary material for: Opioid-Free Anesthesia in Perioperative Care: Findings From a Swedish Web-Based Survey
Source: Pain Res Manag. 2025 Nov 20;2025:6677904. doi: 10.1155/prm/6677904 (PMC12660620; doi:10.1155/prm/6677904)
Supplement: Supporting Information — Additional supporting information can be found online in the Supporting Information section. [file 6677904.f1.docx]

**Supplementary File 1. OFA questionnaire**

This file contains the questionnaire used to assess healthcare professionals' knowledge, attitudes, and practices related to OFA for pain management. The questionnaire includes demographic information, general questions about perioperative care, specific questions on OFA usage and an open-ended question.

1. **Demographic information**
   1. Sex: [ ] Male [ ] Female
   2. Age

[ ] 20-30 years

[ ] 31-40 years

[ ] 41-50 years

[ ] 51-60 years

[ ] > 61 years

- 1. Profession

[ ] Anesthesiologist

[ ] Nurse anesthetist

[ ] Critical care nurses

[ ] Registered nurse with basic education

- 1. Workplace

[ ] University hospital

[ ] County hospital

[ ] Other Hospital/infirmary 

- 1. Professional experience in perioperative practice

[ ] 0-2 years

[ ] 3-6 years

[ ] 7-11 years

[ ] 12-14 years

[ ]  > 15 years

1. **General questions about about intraoperative care**
   1. I am satisfied with the current approach for managing painful stimuli intraoperatively.

[ ] Strongly disagree

[ ] Disagree

[ ] Agree

[ ] Strongly agree

[ ] Don’t know

- 1. I believe that opioids are an essential analgesic component for managing painful stimuli intraoperatively.

[ ] Strongly disagree

[ ] Disagree

[ ] Agree

[ ] Strongly agree

[ ] Don’t know

- 1. There is evidence supporting the use of opioid-free alternatives for managing painful stimuli intraoperatively.

[ ] Strongly disagree

[ ] Disagree

[ ] Agree

[ ] Strongly agree

[ ] Don’t know

- 1. In clinical practice, I primarily use opioid-free medications to manage painful stimuli intraoperatively.

[ ] Strongly disagree

[ ] Disagree

[ ] Agree

[ ] Strongly agree

[ ] Don’t know

1. **Specific questions on opioid-free anesthesia usage**
   1. I have the knowledge to apply OFA for general anesthesia

[ ] Yes [ ] No [ ] Don’t know

**If Yes:**

- - 1. I have acquired knowledge about OFA through

[ ] University lectures

[ ] Workplace lectures

[ ] Scientific articles

[ ] Clinical practice: at least once a week

[ ] Clinical practice: at least once a month

[ ] Clinical practice: at least once a year

[ ] Other sources 

- 1. I believe that OFA is appropriate for the following conditions:

(1 = Strongly disagree, 2 = Disagree, 3 = Agree, 4 = Strongly agree, Don’t know)

- - 1. Patients at risk for or with known history of pain

[ ] 1 [ ] 2 [ ] 3 [ ] 4  [ ] Don’t know

- - 1. Patients at risk for patients at risk for increased opioid consumption or misuse

[ ] 1 [ ] 2 [ ] 3 [ ] 4  [ ] Don’t know

- - 1. Patients at risk for patients at risk for postoperative nausea and vomiting (PONV)

[ ] 1 [ ] 2 [ ] 3 [ ] 4  [ ] Don’t know

- - 1. Patients at risk for reduced postoperative respiratory function

[ ] 1 [ ] 2 [ ] 3 [ ] 4  [ ] Don’t know

- - 1. Patients with malignant disease undergoing cancer surgery

[ ] 1 [ ] 2 [ ] 3 [ ] 4  [ ] Don’t know

- 1. I am interested in developing theoretical knowledge about OFA

[ ] Strongly disagree

[ ] Disagree

[ ] Agree

[ ] Strongly agree

[ ] Don’t know

- 1. I am interested in developing practical skills about OFA

[ ] Strongly disagree

[ ] Disagree

[ ] Agree

[ ] Strongly agree

[ ] Don’t know

- 1. I apply OFA for general anesthesia

[ ] Yes [ ] No [ ]

**If Yes:**

- - 1. The following factors influence my use of OFA for general anesthesia:

1. = Strongly disagree, 2 = Disagree, 3 = Agree, 4 = Strongly agree, Don’t know)
2. The patient’s preference

[ ] 1 [ ] 2 [ ] 3 [ ] 4  [ ] Don’t know

1. ASA Classification

[ ] 1 [ ] 2 [ ] 3 [ ] 4  [ ] Don’t know

1. My professional experience with OFA

[ ] 1 [ ] 2 [ ] 3 [ ] 4 [ ] Don’t know

1. My confidence in performing OFA

[ ] 1 [ ] 2 [ ] 3 [ ] 4 [ ] Don’t know

1. Scientific evidence

[ ] 1 [ ] 2 [ ] 3 [ ] 4 [ ] Don’t know

1. Availability of opioid-free pharmacological alternatives

[ ] 1 [ ] 2 [ ] 3 [ ] 4 [ ] Don’t know

1. Guidelines

[ ] 1 [ ] 2 [ ] 3 [ ] 4 [ ] Don’t know

1. Leadership that supports the use of OFA

[ ] 1 [ ] 2 [ ] 3 [ ] 4 [ ] Don’t know

1. Norms and workplace culture

[ ] 1 [ ] 2 [ ] 3 [ ] 4  [ ] Don’t know

1. The availability of colleagues with knowledge and skills regarding OFA

[ ] 1 [ ] 2 [ ] 3 [ ] 4  [ ] Don’t know

1. Workload and time management

[ ] 1 [ ] 2 [ ] 3 [ ] 4 [ ] Don’t know

- 1. There are guidelines at my clinic supporting the practice of OFA for general anesthesia

[ ] Yes [ ] No [ ] Don’t know

- - 1. I am satisfied with the clinic's guidelines supporting OFA for general anesthesia

[ ] Strongly disagree

[ ] Disagree

[ ] Agree

[ ] Strongly agree

[ ] Don’t know

- - 1. Guidelines regarding OFA for general anesthesia should be implemented in my clinic

[ ] Strongly disagree

[ ] Disagree

[ ] Agree

[ ] Strongly agree

[ ] Don’t know

1. **Is there anything more you would like to add? (open-ended response)**
